# *rstB* Regulates Expression of the *Photobacterium damselae* subsp. *damselae* Major Virulence Factors Damselysin, Phobalysin P and Phobalysin C

**DOI:** 10.3389/fmicb.2017.00582

**Published:** 2017-04-10

**Authors:** Mateus S. Terceti, Amable J. Rivas, Laura Alvarez, Manuel Noia, Felipe Cava, Carlos R. Osorio

**Affiliations:** ^1^Departamento de Microbioloxía e Parasitoloxía, Instituto de Acuicultura, Universidade de Santiago de CompostelaSantiago de Compostela, Spain; ^2^Department of Molecular Biology and Laboratory for Molecular Infection Medicine Sweden, Umeå Centre for Microbial Research, Umeå UniversityUmeå, Sweden; ^3^Departamento de Bioloxía Funcional, Facultade de Bioloxía - CIBUS, Universidade de Santiago de CompostelaSantiago de Compostela, Spain

**Keywords:** RstAB, hemolysin, *Photobacterium damselae*, damselysin, phobalysin, vibriosis, CarSR

## Abstract

The marine pathogenic bacterium *Photobacterium damselae* subsp. *damselae* causes septicemia in marine animals and in humans. The pPHDD1 plasmid-encoded hemolysins damselysin (Dly) and phobalysin P (PhlyP), and the chromosome-encoded hemolysin phobalysin C (PhlyC) constitute its main virulence factors. However, the mechanisms by which expression of these three hemolysins is regulated remain unknown. Here we report the isolation of a mini-Tn*10* transposon mutant which showed a strong impairment in its hemolytic activity. The transposon disrupted a putative sensor histidine kinase gene *vda_000600* (*rstB*), which together with *vda_000601* (*rstA*) is predicted to encode a putative two-component regulatory system. This system showed to be homologous to the *Vibrio cholerae* CarSR/VprAB and *Escherichia coli* RstAB systems. Reconstruction of the mutant by allelic exchange of *rstB* showed equal impairment in hemolysis, and complementation with a plasmid expressing *rstAB* restored hemolysis to wild-type levels. Remarkably, we demonstrated by promoter expression analyses that the reduced hemolysis in the *rstB* mutant was accompanied by a strong decrease in transcription activities of the three hemolysin genes *dly* (damselysin), *hlyA_pl_* (phobalysin P) and *hlyA_ch_* (phobalysin C). Thus, RstB, encoded in the small chromosome, regulates plasmid and chromosomal virulence genes. We also found that reduced expression of the three virulence genes correlated with a strong decrease in virulence in a sea bass model, demonstrating that RstB constitutes a master regulator of the three *P. damselae* subsp. *damselae* hemolysins and plays critical roles in the pathogenicity of this bacterium. This study represents the first evidence of a direct role of a RstAB-like system in the regulation of bacterial toxins.

## Introduction

*Photobacterium damselae* subsp. *damselae* is a primary pathogen of a wide range of marine animals, including cetaceans, crustaceans, mollusks, reptiles and, most frequently, cultivated fish of economic importance in marine aquaculture (Fouz et al., 1992; Rivas et al., 2013b; Terceti et al., 2016). In addition, this pathogen can cause a severe necrotizing fasciitis in humans (Clarridge and Zighelboim-Daum, 1985; Yamane et al., 2004; Hundenborn et al., 2013). The route of entry of *P. damselae* subsp. *damselae* into animal hosts remains poorly investigated. There is sound evidence that water transmits the disease (Fouz et al., 2000), and skin injuries precede *P. damselae* subsp. *damselae* infections (Rivas et al., 2013b). Interestingly, it is known by experimental infection studies that only those strains virulent by the intraperitoneal infection route are also infective through water (Fouz et al., 2000). Previous studies demonstrated that the most virulent *P. damselae* subsp. *damselae* isolates were highly hemolytic and inflicted more severe hemorrhages in diseased fish (Fouz et al., 1992), thus setting a relation between hemolysis and virulence. Later, we discovered that highly hemolytic strains harbor the 153 kb virulence plasmid pPHDD1, which carries the hemolysin genes *dly* and *hlyA_pl_* (Rivas et al., 2011). *dly* gene encodes damselysin, a phospholipase-D active against sphingomyelin (Kreger et al., 1987). *hlyA_pl_* encodes the recently characterized pore-forming toxin phobalysin P (PhlyP) which stands for “photobacterial lysin encoded on a plasmid.” This toxin forms small membrane pores in the target cells causing efflux of K^+^ and entry of vital dyes (Rivas et al., 2015b). In addition, all the hemolytic strains contain *hlyA_ch_* gene in chromosome I (Rivas et al., 2013a, 2014). *hlyA_ch_* encodes phobalysin C (PhlyC), which shares 92% identity in its amino acid sequence with PhlyP. All three hemolysins are known to be secreted via type II secretion system (Rivas et al., 2015a). PhlyP and PhlyC exert an additive effect in hemolysis and virulence, whereas all three hemolysins act synergistically on erythrocytes, a phenomenon that accounts for maximum virulence in mice and fish (Rivas et al., 2013a). Recently, we have demonstrated that hemolysins increase bacterial adherence (Rivas et al., 2015b), which may be of paramount relevance in the early events of natural infection. In addition, tissue damage caused by hemolysins may provide a source of nutrients to further support bacterial infection.

Although the molecular basis of virulence in this pathogen has been extensively studied in recent years, little is known about how the expression of the three hemolysins is regulated. In order to produce virulence factors when required, pathogens must sense environmental changes such as osmolarity, temperature, nutrients and metal ions concentration among others, and thus regulate virulence genes accordingly. It is expected that *P. damselae* subsp. *damselae*, a bacterial pathogen that also possesses a free-living style, has such sensing systems to control hemolysin production.

Two-component regulatory systems (TCS) are particularly important in regulating gene expression in response to environmental signals. A typical TCS consists of a sensor histidine kinase (HK) and its cognate DNA-binding response regulator (RR) (Stock et al., 2000). Upon activation in response to a specific signal, the HK autophosphorylates a conserved histidine residue and this phosphoryl group will be transferred to the conserved aspartic acid of the cognate RR. Phosphorylation of the RR activates an output domain that can modulate gene expression (Gao et al., 2007). Most RRs are transcriptional factors, and once phosphorylated they bind to target promoters, activating or repressing transcription (Stock et al., 2000). Although several TCS are predicted to be encoded in the genome of *P. damselae* subsp. *damselae*, to date none of such systems has been characterized, and their possible role in the regulation of virulence factors is so far unknown.

In this study, we transposon-mutagenized *P. damselae* subsp. *damselae* strain RM-71 and screened for hemolytic defective clones. We found a mutant in the putative sensor histidine kinase gene *rstB*, one of the two genes of the *rstAB* operon that is predicted to encode a TCS, which was strongly impaired in hemolytic activity. Here, we demonstrate that *rstB* is involved in the transcriptional regulation of the plasmid genes *dly* and *hlyA_pl_* and of the chromosomal gene *hlyA_ch_*, and is essential for maximum hemolytic activity. The major role of *rstB* in the virulence of *P. damselae* subsp. *damselae* for fish is also demonstrated. This study represents the first report of a regulatory system of the *P. damselae* subsp. *damselae* virulence. In addition our results constitute the first evidence of the role of a RstAB-like system in regulation of bacterial toxins.

## Materials and Methods

### Bacterial Plasmids, Strains, and Culture Conditions

The bacterial strains and plasmids used in this study are listed in **Table 1**. *P*. *damselae* subsp. *damselae* cells were grown at 25°C on tryptic soy agar (TSA) and broth (TSB) supplemented with NaCl up to 1% (TSA-1 and TSB-1, respectively) and supplemented with antibiotics when appropriate. For hemolysis assays on sheep blood agar plates (Oxoid), strains were cultured on TSA-1 plates and single colonies were inoculated on a blood agar plate, and hemolysis haloes were photographed after 15 h of growth at 25°C. *E. coli* was grown at 37°C in Luria-Bertani (LB) broth or LB agar. When necessary, antibiotics were used at the following final concentrations: kanamycin (Km) at 50 μg mL^-1^, gentamicin (Gm) at 15 μg mL^-1^, chloramphenicol (Cm) at 20 μg mL^-1^. For growth curves, at least three replicates per strain were grown in two independent experiments in 200 μl medium in a 96 well plate inoculated 1:100 from exponentially growing precultures (OD_600_∼0.02) and analyzed using a Biotek plate reader at 10 min intervals.

**Table 1:** Strains and plasmids used and constructed in this study.

Strain or plasmid	Description^a^	Reference/Source
**Strains**		
*P. damselae* subsp. *damselae*		
RM-71	Isolated from turbot; pPHDD1	Fouz et al., 1992
RM-71 *rstB::Tn10*	RM-71 with mini-Tn*10* disrupting *rstB* gene; Km^r^	This study
MT151	RM-71 with in-frame deletion of *rstB* gene	This study
MT157	MT151 with prstAB (complemented mutant); Cm^r^	This study
*E. coli*		
DH5α	Cloning strain	Laboratory stock
S17-1 λ*pir*	RP4-2(Km::Tn7, Tc::Mu-1) *pro-82* λ*pir recA1 endA1 thiE1 hsdR17 creC510*	Herrero et al., 1990
β-3914	F^-^ RP4-2-Tc::Mu Δ*dapA*::(*erm-pir*) *gyrA462 zei-298*::Tn*10* (Km^r^ Em^r^ Tc^r^)	Le Roux et al., 2007
**Plasmids**		
pLOFKm	Tn*10*-based delivery plasmid; Km^r^	Herrero et al., 1990
pMRB24	Cloning vector, *mob*; Cm^r^	Le Roux et al., 2011
prstAB	pMRB24 with *rstAB* genes cloned; Cm^r^	This study
pHRP309	*lacZ* reporter plasmid, *mob* Gm^r^	Parales and Harwood, 1993
pAJR45	*hlyA_ch_* promoter fused to promoterless *lacZ* gene in pHRP309, Gm^r^	Rivas et al., 2013a
pAJR51	*dly* promoter fused to promoterless *lacZ* gene in pHRP309, Gm^r^	Rivas et al., 2013a
pAJR53	*hlyA_pl_* promoter fused to promoterless *lacZ* gene in pHRP309, Gm^r^	Rivas et al., 2013a
pNidKan	Suicide vector derived from pCVD442; Km^r^	Mouriño et al., 2004

^a^*Km^*r*^, kanamycin resistance; Cm^*r*^, chloramphenicol resistance; Gm^*r*^, gentamycin resistance*.

### Mini-Tn*10* Mutagenesis and Identification of the Disrupted Gene

Mini-Tn*10* mutagenesis was performed using the suicide conjugative plasmid pLOFKm (Herrero et al., 1990), with minor modifications as previously described (Rivas et al., 2015a). Genomic DNA from the clones with impaired hemolysis of strain RM-71 was purified with the genome DNA kit (Qbiogene), partially digested with *Bfu*C1 and ligated to *Bam*HI-digested plasmid pUC118. Ligation reactions were transformed into *E. coli* DH5α by electroporation (2.5 kV, 25 μF capacitance, and Pulse Controller Unit set to 200 Ω). Transformants were selected on LB agar plates supplemented with kanamycin and ampicillin. Inserts containing the kanamycin resistance gene of mini-Tn*10* plus flanking chromosomal DNA, were amplified by PCR and sequenced. DNA sequences were obtained using a capillary DNA Sequencer ABI 3730xl (Applied Biosystems). The nucleotide sequence of the *rstAB* loci in RM-71 strain is available in the partially annotated whole genome shotgun sequence of this strain (GenBank Acc. No. LYBT01000056). Locus tags for *rstA* and *rstB* genes are, respectively, A0J47_03465 and A0J47_03460.

### Mutant Construction and Gene Complementation

A non-polar deletion of *rstB* was constructed by allelic exchange using the Km^r^ suicide vector pNidKan as previously described (Rivas et al., 2015a), yielding the *P*. *damselae* subsp. *damselae* mutant strain MT151 (**Table 1**). For complementation of the *rstB* mutant, *rstAB* ORFs sequence together with the respective promoter sequence was amplified by PCR using Hi-Fidelity Kapa Taq, cloned into the Cm^R^ mobilizable vector pMRB24 and mobilized from *E. coli* S17-1-λ*pir* into the MT151 mutant, to yield complemented strain MT157.

### Hemolytic Assays With Bacterial ECPs

To obtain the extracellular products (ECPs), cultures of *P. damselae* subsp. *damselae* in TSB-1 were adjusted to an OD_600_ of 1, and 100 μl were spread with a sterile cotton swab over TSA-1 plates covered with cellophane, as previously described (Liu, 1957). Cells were incubated at 25°C for 48 h and washed off the cellophane using saline solution (0.85% [wt/vol] NaCl) and adjusted to an OD_600_ of 1. Cells were centrifuged at 15,000 ×*g* for 5 min and the supernatants were filtered through 0.22 μm-pore-size membranes and stored at -20°C until used. Quantitative hemolytic assays were carried out in triplicates using the method described by Bernheimer (1988), introducing minor modifications as previously described (Rivas et al., 2015a).

### *lacZ* Transcriptional Fusions and β-Galactosidase Assays

DNA fragments corresponding to *dly, hlyA_pl_* and *hlyA_ch_* presumptive promoter regions extending from about 1 kb upstream of the ATG start codon to about 30 bp downstream of the start codon, were PCR-amplified and fused to a promoterless *lacZ* gene in the low-copy number reporter vector pHRP309. The transcriptional fusions *dly*::*lacZ* (pAJR51), *hlyA_pl_*::*lacZ* (pAJR53) and *hlyA_ch_*::*lacZ* (pAJR45), obtained in a previous study (Rivas et al., 2013a) were here mobilized by conjugation from *E. coli* S17-1-λ*pir* into the parental RM-71 strain and into its *rstB* mutant derivative strain MT151. The *P. damselae* subsp. *damselae* strains carrying the promoter-*lacZ* fusion vectors were grown in TSB-1 and the β-galactosidase activities were measured by the method of Miller (1992). Three independent experiments of β-galactosidase activity measurement were carried out.

### Optical Microscopy

Phase contrast microscopy was performed using stationary phase cultures. Bacteria were immobilized on LB pads containing 1% agarose. Image acquisition was performed using a Zeiss Axio Imager.Z2 microscope equipped with a Plan-Apochromat 63X phase contrast objective lens and an ORCA-Flash 4.0 LT digital CMOS camera (Hamamatsu), using the Zeiss Zen Blue software. For image processing and analysis the MicrobeJ plugin for Fiji was used (Ducret et al., 2016). Cell length and width from 570 to 700 cells per strain (three fields of 190–250 cells each) were measured and statistical significance was calculated using a *t*-test (unpaired).

### Polymyxin B MIC Assay

To determine the polymyxin MIC of RM-71 and the *rstB* mutant MT151, strains were incubated 24 h at 25°C on TSA-1 plates in the presence of *E*-test gradient polymyxin B strips (bioMérieux).

### Fish Virulence Assays

To test the influence of *rstB* gene deletion in the virulence of *P. damselae* subsp. *damselae* for fish, we conducted experimental infection challenges using sea bass (*Dicentrarchus labrax*) as a model, as previously described (Terceti et al., 2016). Fish were obtained from IGAFA (Illa de Arousa, Galicia, Spain). Groups of 10 fish (6 ± 1.2 g) per strain tested and per dose were acclimated in 100 l aquaria at 24°C for 1 week before the infections were performed. The virulence tests were conducted by intraperitoneal injection of bacterial suspensions. Fish were inoculated with 0.1 ml of bacterial suspensions of each strain in 0.85% NaCl solution at two different doses of 10^4^ and 10^3^ CFU/fish. As a control, a group of 10 fish was inoculated with 0.1 ml of sterile 0.85% NaCl solution. Fish mortality was recorded daily for 10 days post-challenge. Re-isolation and identification of the bacteria from the kidney of dead fish were performed. For this purpose, fish were aseptically dissected, kidney samples taken with sterile loops and seeded on TSA-1 and TCBS agar plates. Colonies were confirmed by the subsp. *damselae*-specific *ureC* gene PCR test as previously described (Osorio et al., 2000). The protocols of animal experimentation used in this study have been reviewed and approved by the Animal Ethic Committee of the Universidade de Santiago de Compostela.

## Results

### Transposon Mutagenesis Identifies a *rstAB*-like Operon in *P. damselae* subsp. *damselae* With a Role in Hemolytic Activity

**FIGURE 1:**
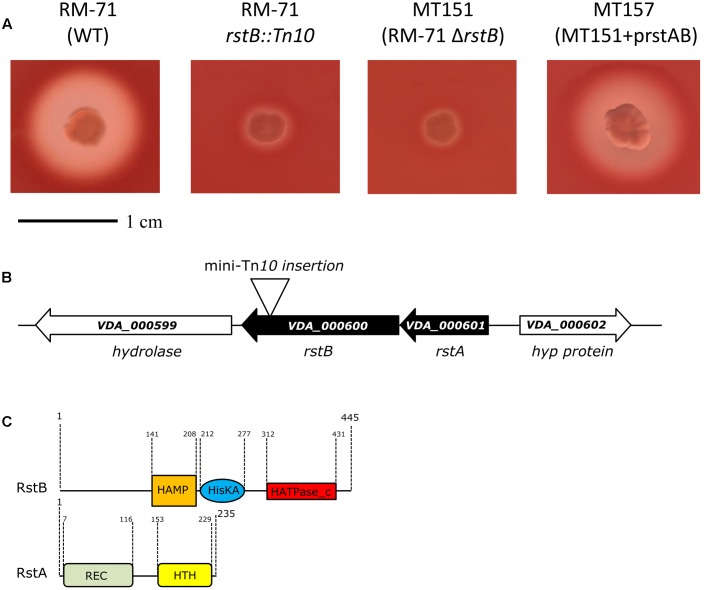
**(A)** Hemolytic phenotypes on sheep blood agar plates of *P. damselae* subsp. *damselae* parental strain RM-71 and derivatives. A mini-Tn*10* transposon mutant in *rstB* gene (RM-71 *rstB*::Tn*10*) shows a reduction in hemolysis, a phenotype which is reproduced in the allelic-exchange Δ*rstB* mutant (MT151). Complementation of MT151 mutant with a plasmid expressing *rstAB* genes (strain MT157) restores the hemolytic phenotype to levels similar to the parental strain. Scale bar, 1 cm. **(B)** Physical map of the *P. damselae* subsp. *damselae rstAB* genes and flanking sequences, with the detailed point of insertion of the mini-Tn*10* transposon in *rstB* gene. Gene numbers refer to gene annotation of the *P. damselae* subsp. *damselae* type strain ATCC 33539 complete genome (GenBank accession no. ADBS00000000). **(C)** Conserved domains of RstB and RstA proteins, showing the characteristic domains of bacterial two-component regulatory systems. RstB is the sensor histidine kinase, and RstA is the response regulator.

In order to gain an insight into how *P. damselae* subsp. *damselae* regulates its hemolytic activity, we screened a library of >2000 mini-Tn*10* insertional mutants in the highly hemolytic and highly virulent pPHDD1-containing strain RM-71, and isolated a total of 12 mutants displaying severely affected hemolytic phenotypes. By genetic analyses we found that 11 out of the 12 mutants had the mini-Tn*10* inserted either in one of the three hemolysin genes or in genes of the previously described type II secretion system that participates in hemolysin secretion (Rivas et al., 2015a), thus validating our screening method (data not shown). Interestingly, we found that the insertion in the remaining mutant clone (RM-71 *rstB*::Tn*10*) with a severely impaired hemolysis (**Figure 1A**) took place in a gene which was 100% identical to the *vda_000600* locus of the *P. damselae* subsp. *damselae* type strain ATCC33539 (GenBank accession number ADBS00000000). This ORF, located in the small chromosome (ChrII) of *P. damselae* subsp. *damselae*, is predicted to constitute a putative two-gene operon together with the upstream gene *vda_000601* (**Figure 1B**).

An *in silico* search for VDA_000600 and VDA_000601 orthologs in other *Vibrionaceae* species showed the highest identity with two ORFs encoded in chromosome I of *Vibrio cholerae* N16961, annotated as VC1319 (50% identity with VDA_000600) and VC1320 (65% identity with VDA_000601), a sensor histidine kinase and a DNA-binding RR, respectively. These two *V. cholerae* ORFs were coined with different names in two independent studies, CarSR (Bilecen and Yildiz, 2009) and VprAB (Herrera et al., 2014), respectively.

Among the *Enterobacteriaceae*, VDA_000600 showed its best match (32% identity) to *E. coli* RstB, and VDA_000601 best match (41% identical) was to *E. coli* DNA-binding RR RstA. The RstAB two-component system is part of the Mg^+2^-sensing PhoPQ regulon of *E. coli* (Ogasawara et al., 2007) and *Salmonella enterica* (Perez et al., 2009). Due to the duality of gene nomenclature in *V. cholerae*, and since most studies on this TCS were conducted with *E. coli* and *S. enterica*, we dubbed the *P. damselae* subsp. *damselae* genes following the original *E. coli* nomenclature. Therefore, VDA_000600 encodes RstB and VDA_000601 encodes RstA in *P. damselae* subsp. *damselae*.

We found that the *P. damselae* subsp. *damselae rstA* stop codon and the *rstB* translational initiation codon are separated by 14 bp, suggesting that they are organized in an operon where *rstA* is the first gene. *rstB* is predicted to encode a 445-amino-acid protein and contains three domains (**Figure 1C**): a histidine kinase-adenylyl cyclase-methyl-accepting protein and phosphatase domain (HAMP), a phosphoacceptor histidine kinase domain (HisKA), and an ATPase domain (HATPase_c). *rstA* is predicted to encode a 235-amino-acid protein and contains two domains: a signal receiver domain (REC) that includes the phosphoacceptor site, and a helix-turn-helix domain (HTH) for DNA-binding. We found that the *P. damselae* subsp. *damselae* RstB and RstA proteins contain the conserved Histidine 222 (H_222_) and Aspartate 55 (D_55_) residues, respectively, which correspond to the sites for phosphorylation in the *V. cholerae* homologs (Herrera et al., 2014) (Supplementary Figure S1). Altogether, the comparative analysis clearly suggests that *P. damselae* subsp. *damselae vda_000600* and *vda_000601* genes encode a RstAB-like TCS.

### *rstB* Deletion Mutants Are Impaired in Hemolytic Activity

**FIGURE 2:**
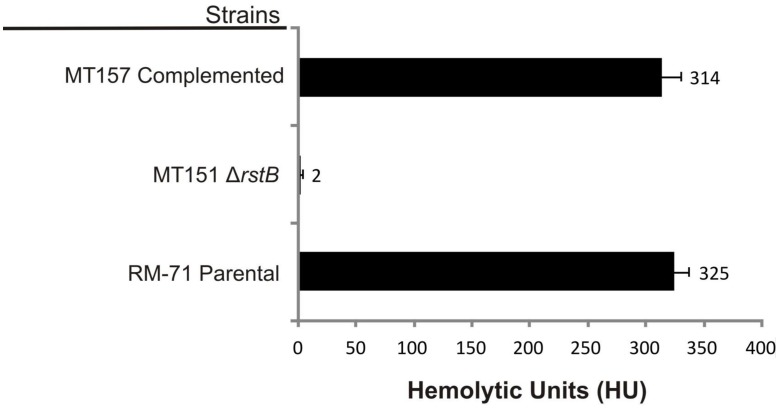
**Hemolytic activities of parental strain RM-71, *rstB* deletion mutant MT151, and complemented strain MT157 (prstAB), determined with bacterial extracellular products (ECPs) and with suspensions of sheep erythrocytes**. The *rstB* mutant is severely impaired in hemolysis, with a reduction of hemolytic units (HU) from 325 to 2 HU. The release of hemoglobin was determined at A_540_. One hemolytic unit is defined as the amount of hemolysin which lyses 50% of sheep erythrocytes. All assays were carried out in triplicate and mean values with standard deviation are shown.

To confirm the association between the transposon mutation of *rstB* and the impaired hemolysis, a non-polar deletion of *rstB* was generated, yielding strain MT151. As a result, deletion of *rstB* caused the same hemolytic phenotype observed in the transposon mutant (**Figure 1A**). In order to obtain a quantitative measure of the role of *rstB* in the hemolytic activity, assays with sheep erythrocyte suspensions and bacterial ECPs were conducted. As a result, we found that deletion of *rstB* caused a decrease of the hemolytic activity from 325 hemolytic units (HU) produced by the parental (RM-71) to 2 HU of the Δ*rstB* mutant (MT151) (**Figure 2**). In order to complement the *rstB* mutant, we cloned the complete *rstAB* operon with its putative promoter sequence upstream *rstA* into the mobilizable, Cm^R^ plasmid pMRB24, yielding plasmid prstAB. We found that complementation of MT151 with this plasmid (yielding strain MT157) fully restored the hemolytic halo (**Figure 1A**) and HU of the parental strain (**Figure 2**). These results altogether clearly indicate that *rstB* plays a major role in the hemolytic activity in *P. damselae* subsp. *damselae.*

### Expression of *dly*, *hlyA_pl_* and *hlyA_ch_* Genes Is Controlled by RstB

**FIGURE 3:**
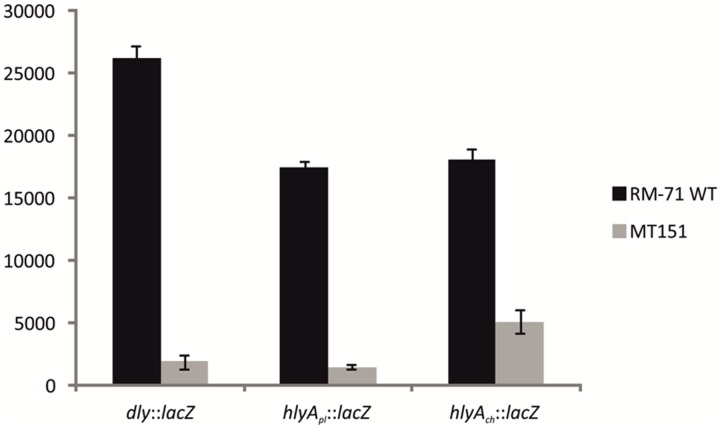
**Transcriptional activity (*β*-galactosidase units) of the *lacZ* fusions to the *dly*, *hlyA_pl_*, and *hlyA_ch_* promoters, under parental (RM-71) and Δ*rstB* mutant (MT151) backgrounds**. All experiments were carried out in triplicate. Mean values with standard deviations denoted by error bars are shown.

**FIGURE 4:**
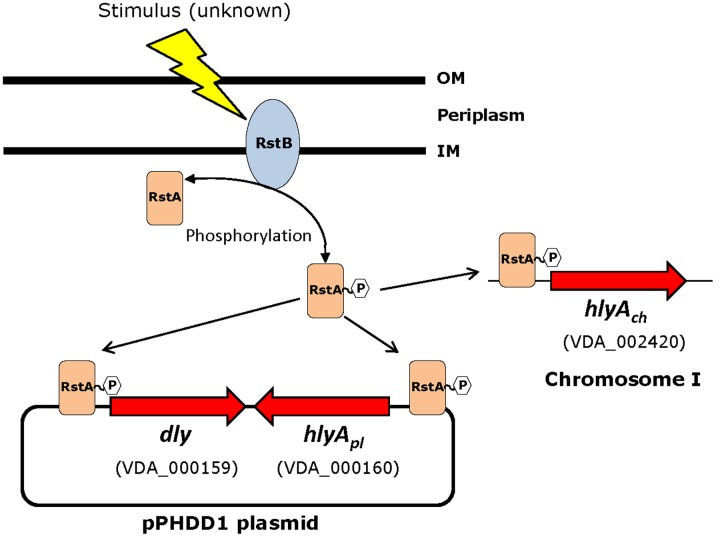
**Schematic depicting the three hemolysin genes reported in this study to be part of the RstAB regulon of *P. damselae* subsp. *damselae***. Note that two hemolysin genes (*dly* and *hlyA_pl_*) are encoded in pPHDD1 plasmid whereas *hlyA_ch_* is encoded in chromosome I. Gene numbers refer to the annotated genome of the type strain ATCC33539.

Based on the above observations that *P. damselae* subsp. *damselae rstB* mutation strongly impairs hemolytic activity, we next examined whether RstB regulates the expression of the three hemolysin genes at the transcriptional level. For this, we evaluated changes in the expression of *dly*, *hlyA_pl_* and *hlyA_ch_* promoters cloned in vector pHRP309 upstream of a promoterless *lacZ* gene. Transcription was measured by determining β-galactosidase activities in parental and mutant strains. We observed that under standard TSB-1 culture conditions, deletion of *rstB* in the pPHDD1-harboring strain RM-71 caused a 15-fold, 10-fold, and 3-fold decrease in β-galactosidase levels of the *dly::lacZ*, *hlyA_pl_::lacZ*, and *hlyA_ch_::lacZ* fusions, respectively (**Figure 3**). These results clearly suggest that the hemolytic defect of the *rstB* mutant is due to an effect on hemolysin gene transcription. It is thus noteworthy that the *rstAB* system, encoded in the chromosome II of *P. damselae* subsp. *damselae*, regulates two plasmid-encoded hemolysins and a chromosome-I encoded hemolysin (**Figure 4**).

A previous study reported that RstA-regulated genes in *E. coli* share a conserved consensus sequence in their promoters, the so-called RstA box (TACATNTNGTTACA) (Ogasawara et al., 2007). We *in silico* analyzed the nucleotide sequences of *dly*, *hlyA_pl_* and *hlyA_ch_* gene promoters for presence of conserved motifs that might act as RstA-binding sequences (Supplementary Figure S2). The three promoters diverged notably in their nucleotide sequences, and even though PhlyP and PhlyC amino acid sequences are 92% identical their respective promoters bear little similarity (Rivas et al., 2013a). Interestingly, we detected a short conserved sequence among the three promoters that shared some residues in common with the *E. coli* consensus RstA box (Supplementary Figure S2).

### The *rstB* Mutant Exhibited Normal Growth and Cell Morphology, and Showed No Detectable Changes in Polymyxin Sensitivity

**FIGURE 5:**
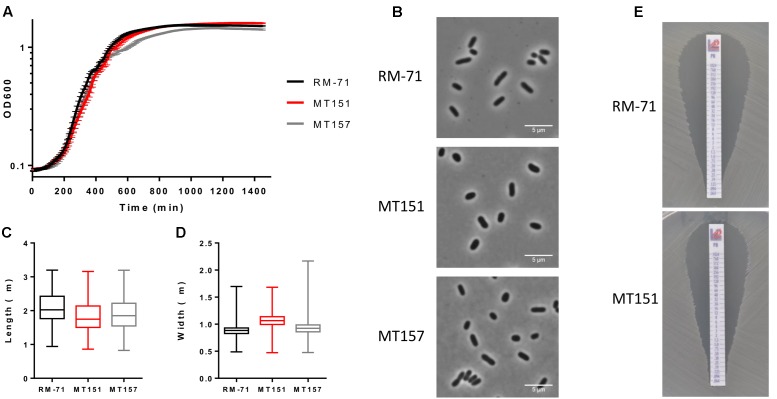
**(A)** Growth curves of RM-71, *rstB* mutant (MT151) and complemented strain (MT157) in TSB-1 medium. **(B)** Phase contrast images of cells grown to stationary phase in TSB-1. Scale bars 5 μm. **(C)** Box plot graphs showing the comparison of cell length in stationary phase cultures. Whiskers indicate min and max values. **(D)** Box plot graphs showing the comparison of cell width in stationary phase cultures. Whiskers indicate min and max values. **(E)** *E*-test for polymyxin sensitivity showed no differences between RM-71 and the *rstB* mutant MT151.

To determine the importance of *rstB* in *P. damselae* subsp. *damselae* physiology beyond its role in hemolysin gene regulation, we analyzed the effect of *rstB* gene deletion on growth. We observed that absence of *rstB* had no apparent effect on growth in TSB-1 medium, as both mutant and complemented cultures reached identical optical densities as the wild-type (**Figure 5A**). Microscopy analysis revealed that there were also no morphological differences -i.e., cell width and length- between these strains (**Figures 5B–D**).

Previous studies reported that *V. cholerae vprAB* mutants (homologs of *P. damselae* subsp. *damselae* RstAB) exhibited sensitivity to the cationic antimicrobial peptide polymyxin B (Herrera et al., 2014; Bilecen et al., 2015). To date, the influence of polymyxin B in *P. damselae* subsp. *damselae* viability had not been explored. In order to test the response of *P. damselae* subsp. *damselae* parental strain and *rstB* mutant to polymyxin B, we determined the MIC for this compound using gradient polymyxin B E-strips in TSA-1 agar medium. We found that both the parental strain RM-71 and the *rstB* mutant MT151 were highly sensitive to polymyxin B, displaying a MIC of 0.125 μg/mL (**Figure 5E**). Contrary to what has been reported in *V. cholerae*, deletion of *rstB* does not influence polymyxin sensitivity in this pathogen.

### RstB Plays a Major Role in Virulence for Fish

**FIGURE 6:**
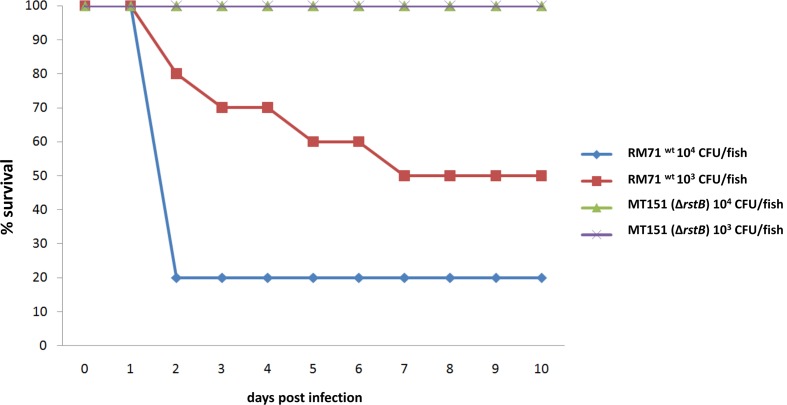
**Survival (%) of sea bass intraperitoneally challenged with two different doses (10^4^ CFU/fish and 10^3^ CFU/fish) of the *P. damselae* subsp**. *damselae* parental strain (RM-71) and the Δ*rstB* mutant MT151. For each dose assayed, a total of 10 fish were inoculated.

In a previous study we demonstrated that *dly*, *hlyA_pl_* and *hlyA_ch_* genes play a key role in the virulence of *P. damselae* subsp. *damselae* in fish (Rivas et al., 2013a). Since we have here demonstrated that *rstB* is involved in the regulation of these three toxin genes, we wanted to evaluate its contribution to *P. damselae* subsp. *damselae* virulence. For this purpose, virulence tests were conducted in sea bass (*Dicentrarchus labrax*) as a model fish, inoculating the parental strain RM-71 as well as its *rstB* derivative mutant MT151. The challenges were conducted in fish kept at a water temperature of 24°C and using two different doses of 10^4^ and 10^3^ CFU/fish, and 10 fish for each dose and strain. The parental strain RM-71 was virulent for sea bass, with mortality rates of 80 and 60% at each dose, respectively (**Figure 6**). Noteworthy, this strain caused the death of 80% of the animals in less than 48 h at the higher dose (10^4^ CFU/fish). On the contrary, the *rstB* mutant strain MT151 did not cause any mortality at the two assayed doses (10^4^ and 10^3^ CFU/fish). *P. damselae* subsp. *damselae* RM-71 could be re-isolated from the kidney of all dead fish post-challenge, and colonies were confirmed by phenotypical tests and by a colony-PCR targeted to the *P. damselae* subsp. *damselae*-specific *ureC* gene (data not shown). These results thus demonstrate that mutation of *rstB* strongly impairs virulence of this pathogen for fish.

## Discussion

Several TCS have been reported to regulate toxin expression in species of the *Vibrionaceae*. This is the case of the *V. cholerae* ToxRS and VarS/VarA systems that enhance the expression of ToxT, a transcriptional factor that positively regulates cholera toxin production (DiRita et al., 1991; Jang et al., 2011). Also, the *V. parahaemolyticus* ToxRS system regulates effector proteins of the type III secretion system (Whitaker et al., 2012).

In this study we identified *rstB*, the predicted histidine-kinase gene of the RstAB-like two-component system that positively regulates the three hemolysin genes of *P. damselae* subsp. *damselae*. To the best of our knowledge this represents the first evidence of a RstAB homologous system that regulates toxin genes in a pathogenic bacterium. The singularity of our findings is reinforced by the fact that one of the regulated genes is in chromosome I (*hlyA_ch_*), whereas the two other regulated genes (*dly* and *hlyA_pl_*) are encoded within a horizontally acquired large virulence plasmid (pPHDD1). It is thus interesting to note that *rstB*, encoded in the small chromosome of *P. damselae* subsp. *damselae*, can both regulate plasmid and chromosomal loci. Homologues of RstAB have received little attention to date, but some knowledge has been gained in the discovery of the RstAB regulon, and specific genes whose expression is regulated directly by the RR RstA have been identified. The RstAB regulon seems to be little predictive: whereas *E. coli* RstAB positively regulates the three genes *yfiA*, *entE* and *cspC* (Oshima et al., 2002), expression of *yfiA* and *cspC* in *S. enterica* remained unaffected both under over-expression of *rstA* and under deletion of *rstA* (Cabeza et al., 2007). A recent study evidenced that a *S. enterica rstB* mutant had reduced expression levels of genes related to pyrimidine metabolism (*udp* and *cdd* genes), enterobactin biosynthesis (*entA*, *entB*, *entE*, and *entF*) and ferrous iron transport (*feoA*, *feoB*) (Tran et al., 2016). These results thus suggest that the RstAB TCS of different species, although phylogenetically closely-related, govern the expression of distinct groups of genes in different bacteria.

Within *Vibrionaceae*, the first evidence for the role of RstAB homologues came after the identification of *V. cholerae* genes VC1319 and VC1320. These genes were dubbed *carS* and *carR* respectively in a study aimed at identifying genes regulated by calcium, and it was determined that CarSR negatively regulated biofilm formation and expression of the *vps* polysaccharide genes (Bilecen and Yildiz, 2009). Later, it was found that *carSR* transcription was negatively regulated in response to an external increase of Ca^2+^, and CarSR negatively regulated *vps* gene expression and biofilm formation (Bilecen et al., 2015). In another study, the same *V. cholerae* genes alternatively dubbed VprB/VprA, were found to regulate expression of the *almEFG* operon, which encodes proteins necessary for glycine modification of lipid A and required for colonization of a mammalian host (Herrera et al., 2014). This addition of glycine residues to the lipid A domain of lipopolysaccharide conferred polymyxin resistance. We have demonstrated here that the *P. damselae* subsp. *damselae rstB* mutant exhibits the same sensitivity to the cationic antimicrobial peptide polymyxin as the parental strain. Since no homologs of *almEFG* genes are found in the complete genome of the *P. damselae* subsp. *damselae* reference strain ATCC33539, it is likely that *P. damselae* subsp. *damselae* does not share the lipid A modification function with *V. cholerae*.

The conservation of the candidate phosphorylation sites of RstB_H222_ and RstA_D55_, coincident with the same conserved residues in their RR and histidine kinase orthologs (Gao and Stock, 2009; Herrera et al., 2014) (Supplementary Figure S1) support the consideration of RstAB as the first putative TCS identified to date in *P. damselae*. We demonstrated here that RstB is necessary for optimal expression of the *dly*, *hlyA_pl_* and *hlyA_ch_* genes, and this regulation is likely exerted at the transcriptional level. Although the PhlyP and PhlyC amino acid sequences are 92% identical, the nucleotide sequences of their respective promoters bear little similarity (Rivas et al., 2013a). Still, we were able to detect a putative consensus sequence common to the three hemolysin gene promoters that might constitute a candidate for RstA binding (a RstA box) (Supplementary Figure S2), and shared similarity to the consensus RstA box of *E. coli* (Ogasawara et al., 2007). Evidences with other bacterial species revealed that the RstAB two-component system regulates gene functions by acting at the transcriptional level. This is the case of *E. coli*, where RstA is known to bind to a narrow region of 200 bp upstream the *csgD* promoter and regulate its transcription (Ogasawara et al., 2010). In addition, RstAB homologs can exert regulatory functions acting at other levels, as is the case of *Salmonella enterica*, where RstA promoted a reduction of RpoS cellular levels by inducing RpoS degradation (Cabeza et al., 2007).

The three hemolysin genes regulated by RstAB play a crucial role in virulence of *P. damselae* subsp. *damselae* for mammals and for fish and are responsible for hemolytic and cytotoxic activity (Kreger et al., 1987; Rivas et al., 2013a, 2015b). We have shown here that deletion of *rstB* strongly impairs virulence in the sea bass fish model. To date, only a few reports have demonstrated a direct role of RstAB in virulence. A *Yersinia pseudotuberculosis rstA* mutant was less virulent than the parental strain for mice (Flamez et al., 2008), but the potential virulence factors that are regulated by *rstAB* remain unknown. In *V. cholerae*, VprAB were found to be necessary for optimal intestinal colonization in a suckling mouse model, likely due to the role of VprAB as regulators of lipidA modification (Herrera et al., 2014). Lately, it was reported that *rstAB* mutations reduced virulence of avian pathogenic *E. coli* in a bird animal model (Gao et al., 2015), but these authors did not identify which virulence genes were regulated by RstA.

Expression of the *rstAB* genes is known to be under the control of the Mg^2+^-sensing PhoQP two-component system in *E. coli* (Ogasawara et al., 2007) and in *Salmonella enterica* (Perez et al., 2009). However, the specific stimuli that trigger the activation (autophosphorylation) of the sensor histidine kinase RstB in *Enterobacteriaceae* and of CarS/VpR in *V. cholerae* still remain undeciphered. Herrera et al. (2014) found that *vprA* promoter expression responded to biological signals as bile, acidic pH and sublethal concentrations of polymyxin B, but did not elucidate which signals trigger VprB phosphorylation. Similarly, the stimulus that triggers the RstAB-dependent hemolysin promoter activation in *P. damselae* subsp. *damselae* is so far unknown. *P. damselae* subsp. *damselae* has likely evolved adaptive responses to maximize hemolysin production into the host but not in its free-living planktonic stage. Ongoing studies aimed at elucidating the sensory signals that trigger the RstAB-dependent regulation of the expression of the three major virulence factors in *P. damselae* subsp. *damselae* are expected to reveal novel cues on this fascinating model of pathogenesis.

## Author Contributions

MT, AR, LA, and MN performed the experiments. AR, LA, and FC helped in data analysis and manuscript editing. CO designed and directed the research and wrote the manuscript.

## Conflict of Interest Statement

The authors declare that the research was conducted in the absence of any commercial or financial relationships that could be construed as a potential conflict of interest.
