# Supplementary material for: rstB Regulates Expression of the Photobacterium damselae subsp. damselae Major Virulence Factors Damselysin, Phobalysin P and Phobalysin C
Source: Front Microbiol. 2017 Apr 10;8:582. doi: 10.3389/fmicb.2017.00582 (PMC5385354; doi:10.3389/fmicb.2017.00582)
Supplement: Supplementary file 1 [file Data_Sheet_1.DOCX]

RSTA:

P.DAMS MTETYSLLIIEDDLKLQQMLADYFLTQGFTVQTHSDGNGAIELIESSNPDIVLL**D**LMLPG 60

V.CHOL MSNQPSLYIIEDDTKLREMLAEYMTNQGFQVTTFATGETAPEQILLNQPDLVLL**D**LMLPG 60

E.COLI MNVMNTIVFVEDDAEVGSLIAAYLAKHDMQVTVEPRGDQAEETILRENPDLVLL**D**IMLPG 60

*. :: ::*** :: .::* *: .:.: * . *: * * * .:**:****:****

P.DAMS NDGLTICRQVRGKYQGKLMMLTASNDDFDHVAALEIGADDYVVKPIKPRVLLARMRMLLR 120

V.CHOL ENGLTICRQIRAQFLGKILMLTASDDDFDHVAALEMGADDFVNKPIKPRVLLARIRMLMR 120

E.COLI KDGMTICRDLRAKWSGPIVLLTSLDSDMNHILALEMGACDYILKTTPPAVLLARLRLHLR 120

::*:****::*.:: * :::**: :.*::*: ***:** *:: * * *****:*: :*

P.DAMS RAKIQP-------DTPVNLNVLTYGNLTLHKNRKLCEFSGQKICMTDSEFDLLWLLASSP 173

V.CHOL REERTS-------ASADATHLLQFGGLLLNQSRRHCELDGEVINLSDSEFDLLWLLASAA 173

E.COLI QNEQATLTKGLQETSLTPYKALHFGTLTIDPINRVVTLANTEISLSTADFELLWELATHA 180

: : : : * :* * :. .: : . * :: ::*:*** **:

P.DAMS DAPLSRDFLTKELRGIEYDGIDRTIDNKIVSLRKKLLDDSSTPQRIITVRGKGYLFVPDT 233

V.CHOL DQVVSREFLTKSLRGIEYDGLDRTVDNKIVTLRKKLCDDSSTPKRIITVRGKGYLFVPDT 233

E.COLI GQIMDRDALLKNLRGVSYDGLDRSVDVAISRLRKKLLDNAAEPYRIKTVRNKGYLFAPHA 240

. :.*: * *.***:.***:**::* * ***** *::: * ** ***.*****.*.:

P.DAMS WV 235

V.CHOL W- 234

E.COLI WE 242

*

RSTB:

P.DAMS MRRLYIESFIGLIVLFGLSLYCYEVIIYRWNTDYDYVLEDYEAEALHDLVDNIYHNEGSK 60

V.CHOL MRRIYIESLVSLIVLFFGSLVSYSFIVYELDTDYDYVLEDYQAEALQTLLSQIRQLDSPE 60

E.COLI MKKLFIQFYLLLF-------VCFLVMSLLVGLVYKFTAERAGKQSLDDLMNSSLYLMRSE 53

*::::*: : *: .: .: . *.:. * ::*. *:.. :

P.DAMS AAYLAIEKYATKTRQTLTVHTKDALPEDVKSFFFTTAPQSYTYHDDD------------- 107

V.CHOL SAKTALRGYAEHIHKTVDLLPLTELPPEVQQHFSSEAAHSPIFHDDD------------- 107

E.COLI LREIPPHDWGKTL-KEMDLN----------LSFDLRVEPLSKYHLDDISMHRLRGGEIVA 102

. :. : : : * . :* **

P.DAMS ---RILWFRLTLSSDFYSLKPNFDTPLR--RAIDFDDNMVWVFFMGGFALYSILFIWYLS 162

V.CHOL ---RNLWLQLDSNEQIYLLKPDSHSPVY--QAIDRADNLLFVFMLGGFALYCMFLIWFLS 162

E.COLI LDDQYTFLQRIP-RSHYVLAVGPVPYLYYLHQMRLLDIALIAFI--AISLAFPVFIWMRP 159

: ::: . * * . : : : * : .*: .::* .:**

P.DAMS R--RVRLLEKTTLAFAHGEFAARARTESRFRVGTLNQSFNYMADKISNLLISNKALTNAI 220

V.CHOL R--RLRELERVTHDFANGNLQARASTKSSKSVGKLNHSFNMMADKISHLILSNKALTNAI 220

E.COLI HWQDMLKLEAAAQRFGDGHLNERIHFDEGSSFERLGVAFNQMADNINALIASKKQLIDGI 219

: : ** .: *..*.: * .. . *. :** ***:*. *: *:* * :.*

P.DAMS A**H**ELRTPIFRIQWQAELLADSVKDKEIQQKIESIVEDTEEMESMVNELLYYAKVEQPDTE 280

V.CHOL A**H**DLRTPIFRIQWQAEVLAESQLNAKQQQQVASIIEDTEEMETMVDELLYYAKLEHPESD 280

E.COLI A**H**ELRTPLVRLRYRLEMSDNLSA-----AESQALNRDISQLEALIEELLTYARLDRPQNE 274

**:****:.*:::: *: : : :: .* .::*::::*** **::::*:.:

P.DAMS LYCQDIVFNQYLNDMLQNWEKTAQSKITLKTPFKQNIVSSIDTQLFKHVLNNLVSNASRY 340

V.CHOL IQSQYLEVNQWLADFIDEQSHKSSLQIQY-LPSLQPLMLDADPQLLTRALVNLIRNAEKY 339

E.COLI LHLSEPDLPLWLSTHLADIQAVTPDKTVRIKTLVQGHYAALDMRLMERVLDNLLNNALRY 334

: . . :* : : . : : * * :*: :.* **: ** :*

P.DAMS SRKHILVTLSQERDSVFITVEDDGPGIPNEHWPYLFDPFYSADPARNKAQSGFGLGLAIA 400

V.CHOL AGDRLLIEASTVDAKLCIAIHDNGPGIEEQHWPHIFDAFYSTDSSRNKAQSGFGLGLAIV 399

E.COLI CHSTVETSLLLSGNRATLIVEDDGPGIAPENREHIFEPFVRLDPSRDRSTGGCGLGLAIV 394

. . : : :.*:**** :: ::*: * * :*::: .* ******.

P.DAMS KQIVARHKGQITIGDSALLGGAKFTVILPITCQCDPILDRPNISK 445

V.CHOL KQIMARHQGEVTLTKSP-LGGACFSLWLPLYPNKLELPVNKQ--- 440

E.COLI HSIALAMGGTVNCDTSE-LGGARFSFSWPLWHNIPQFTSA----- 433

:.* * :. * **** *:. *: : :

**Supplementary Figure S1.** Sequence alignment of RstA and RstB proteins from *P. damselae* subsp. *damselae* (P.DAMS) and their homologues from *V. cholerae* (V.CHOL) and *E. coli* (E.COLI). The conserved Aspartate 55 (D_55_) residue in RstA and Histidine 222 (H_222_) residue in RstB, corresponding to the sites for phosphorylation in the *V. cholerae* homologues (Herrera et al., 2014), are highlighted in red.

dly tatcgagatatcaagtacaacctaatattcttaaccatctaaataaaaagataaaaacag

hlyApl ---------------------------------------------tcaaacaggatctca

hlyAch ---------------attaacttattactcattagagtatgaaaaacaaataagatctca

** *

dly atacaaaaaaaa--ttattgtcatacaa-atctaaaca----------------------

hlyApl atagtaaaaaagtaatattgaattgaaaatt-----------------------------

hlyAch atagtaaaaaagtaatatcgaattacaaattctgagcagagaggagatattatttttaat

*** ****** *** * * ** *

dly -----------------------------------------------gaaaacaaaacgg

hlyApl ------------------ttggctgttttgagtttataacttatacagagataaaaacga

hlyAch attggataacgtcacgaagagttagcgatggtctaatcacttatatagagataagaaagg

** * * ** *

dly ataaag---------------cttaacctgccagatagcgta**taggttattagac**ctatc

hlyApl atctattctttggagtaaataattaaataaggatgatctgaa**tacattatttgat**atgag

hlyAch atttattctttggagtaaacatttaaaaagcgatggtctaaa**tacgttatttgac**atgag

** * **** * *** ***** ** *

dly gcg---acttctattacatcaatatttcagttggttata-----------tggacattta

hlyApl aacagaagcattatttaaaaactattgtaata--------------------aaaatgtt

hlyAch agtgtaaacactataacaaaaatatttaaattatttaaaaaatattgtaacgaaaatttt

* *** * * **** * * * ** *

dly ctt**ATG**

hlyApl catatggagacttt**ATG**

hlyAch tatatggggatatt**ATG**

****

**Supplementary Figure S2.** Sequence alignment (Clustal Omega) of *hlyA_pl_*, *hlyA_ch_* and *dly* gene promoters. The ATG translational start codon is highlighted in bold. A conserved sequence in *hlyA_pl_* and *hlyA_ch_* promoters, which bears similarity to the *E. coli* consensus RstA box (TACATNTNGTTACA) is highlighted in bold and coloured in red.
